# Actively tackling inactivity

**DOI:** 10.3399/bjgp22X720497

**Published:** 2022-09-02

**Authors:** Steve Haake, Simon Tobin

**Affiliations:** The Advanced Wellbeing Research Centre, Sheffield Hallam University, Sheffield.; Norwood Surgery, Southport.

## PHYSICAL ACTIVITY GUIDELINES, LIFE EXPECTANCY, AND THE QUEEN

Environments in the UK have been designed to make our lives physically easier: cars, trains, lifts, washing machines — even the humble remote control. It’s no surprise then that around 4 out of 10 of us are inactive.^1^ While many people may know that physical activity is good for them, they struggle to do any. And, while the phrase ‘5-a-day’ for the consumption of fruit and veg is well known, there’s no equivalent for physical activity. Perhaps that’s why only between 8% and 18% of people in the UK were able to recall that the Chief Medical Officers’ guidance is at least 150 minutes of moderate exercise per week.^2^^–^^4^ GPs faired marginally better: just 20% could recall the guidance.^5^

The consequences of inactivity in our population are well known: high levels of cardiovascular disease, diabetes, cancer, and poor mental health. These are self-evident from Fingertips, the public health statistics gateway for general practices in England. Consider, for instance, the under 75 mortality rate from preventable cardiovascular diseases. According to Fingertips, if we look at towns across the country, the mortality rate correlates linearly with inactivity with a correlation coefficient of *r* = 0.67 (moderately strong).^6^ If inactivity increases in a town, so do preventable deaths. Given all the other determinants of health — alcohol, diet, drugs, access to health care, education, employment, environment, and poverty (and so on) — this is quite remarkable.

As you might expect from this, healthy life expectancy (HLE) also correlates with levels of inactivity. Figures from the Office for National Statistics show that HLE for males in the UK is just 62.8 years^7^ — less time than the Queen has been on the throne. Shockingly, only four towns have a HLE at birth for males of ≥70 years: Richmond upon Thames, Rutland, Wokingham, and, of course, Windsor. Notably, they all have activity levels of at least 68% and inactivity levels <21%.^6^

## WHY PEOPLE GET ACTIVE AND STAY ACTIVE

Despite being unable to quote the precise physical activity guidance, a quarter of patients say they would be active if advised to do so by a doctor or nurse.^8^ And yet, in one study of patients who were overweight and obese accessing primary care, 70–80% of professionals didn’t speak to them about physical activity.^9^ Understanding and appreciating the drivers and barriers to becoming more active would help considerably.

One way of learning more is to look at parkrun, on which there is now a plethora of research. Usually written with a small ‘p’, it is a charity that puts on weekly, timed 5 km and junior 2 km events where participants can run, walk, or volunteer (volunteering is considered participation by parkrun). Importantly, parkrun is free.

To promote the health and wellbeing of primary care workers and patients, the Royal College of General Practitioners set up the parkrun practice initiative to link surgeries with their local parkrun:^10^ 4 years later, 1600 have joined (around 20% of all GP practices).^11^^,^^12^ In presentations of our parkrun research to GPs, link workers, and health practitioners, it’s surprising how many perceive parkrun as a lycra-clad race only for the usual sporty types. They don’t see parkrun as appropriate for themselves, never mind their patients.

This led to research in which we stratified a survey of 59 999 adult parkrunners from fastest to slowest to answer the seemingly simple question, ‘what is a parkrunner?’ The quick answer is — it depends where you look.^13^ Those at the front were very different to those at the middle or the back. The front was dominated by skinny young males, keen to win and focused on competition; very few had health conditions. The middle was composed almost equally of men and women, with 8% having long-term health conditions. The back was starkly different to those ahead. Some were doing 5 km for the first time since they’d left school, some were doing couch-to-5k, some were combining running with walking, and some were walking the whole thing and wondering if they could finish. Those at the back were more likely to be women, older, retired, and have health issues. Overall, 45% of walkers had long-term health conditions such as arthritis, depression, anxiety, asthma, and hypertension — precisely the conditions for which GPs say they would recommend physical activity.

We asked parkrunners what motivated them to turn up to their first parkrun and what they perceived as the benefits. The former drives behaviour change while the latter sustains it. As expected, fitness was important as a motive, but slower runners and walkers were more interested in their health, being outdoors, and being active in a safe environment. While few chose improving mental health, happiness, or feeling part of a community as a motive, around 70–80% reported improvements, regardless of finishing time.

## A SIMPLE MESSAGE TO PATIENTS AND STAFF

We need to focus efforts on motivating those at highest risk of disease — those who are inactive or have low levels of activity. These groups want to increase their activity but would like it to be free, community-based, inclusive, safe, and in green spaces. The perception of competitive athletes in shorts and vests can be a major barrier for many.

The parkrun model illustrates a way to motivate people to do activity and participation is increasing, not just in the UK, but in 23 countries across the world. parkrun does better than many interventions when it comes to uptake in groups less likely to be physically active — women, those with health conditions, and those in areas of deprivation.

The community aspect of physical activity really matters to slower runners and walkers, who value the social connections they make. Events like parkrun can bring neighbourhoods together and act to create and then strengthen bonds between people; they are like community adhesive. Physical activity events can also impact directly at a practice level, increasing the resilience of GPs and their teams. Many parkrun practices have organised takeover days that create a social buzz and highlight the value they place on physical activity. Primary care teams can literally talk the talk and walk the walk. Volunteering at events can transform lives and has been shown to add a level of happiness and satisfaction even greater than participating in the physical activity itself.^14^ Social prescribing link workers have a key role in signposting to local physical activity groups.

The general public and healthcare professionals clearly understand the message about physical activity, even if they can’t remember the targets. Perhaps it would help to have a catchy slogan, but *‘moderate physical activity for 30 minutes, 5 times a week’* just won’t do. Maybe instead we should simply ask people to *‘move more, more often’*.
